# Rodent Models of D-Galactose Induction of Accelerated Aging: A Platform for Exploring Kidney Aging Mechanisms and Anti-Kidney Aging Strategies

**DOI:** 10.3390/cells15090766

**Published:** 2026-04-24

**Authors:** Shaona Niu, Ryan S. Azzouz, Liang-Jun Yan

**Affiliations:** Department of Pharmaceutical Sciences, UNT System College of Pharmacy, University of North Texas Health Science Center, Fort Worth, TX 76107, USA; berry_2000@163.com (S.N.); ryanazzouz@my.unthsc.edu (R.S.A.)

**Keywords:** D-galactose induction of aging, apoptosis, kidney aging, oxidative stress, fibrosis, inflammation

## Abstract

Epidemiological studies have demonstrated that kidney aging is a risk factor for acute kidney injury (AKI) and chronic kidney disease (CKD). Therefore, understanding the mechanisms of kidney aging is key to designing novel anti-kidney aging strategies. In this regard, animal models of kidney aging are essential tools. In this review article, we focus on D-galactose (D-gal)-induced accelerated aging in rodents. This animal aging model is a popular and widely used experimental method in the field of aging and aging-related degenerative disorders. It has been shown that the major characteristics of the D-gal-induced aging process are increased oxidative stress, decreased antioxidant enzymes, elevated cell death, increased tissue fibrosis, and accumulation of inflammatory mediators. This review focuses on D-gal-induced kidney aging in mice and rats, with discussions on both kidney aging mechanisms and anti-kidney aging regimens using this model. It is our belief that D-gal induction of accelerated kidney aging will continue to be used as a convenient platform for elucidating kidney aging mechanisms and exploring novel anti-kidney aging targets that may slow down kidney aging and retard the development of aging-related renal disorders.

## 1. Introduction

The kidney is a vital life-sustaining organ that is in charge of blood filtration and waste excretion. It is involved in numerous homeostatic processes, such as blood pressure regulation, pH maintenance, hormone secretion, electrolyte and mineral retention, and the body’s fluid volume regulation [1,2]. Under hypoglycemic conditions, the kidney can also make glucose via the gluconeogenic pathway [3]. Achieving all these functions requires strict maintenance of the nephron’s cellular membrane potential, tight regulation of membrane channels, transporters, and receptors [1]. All such highly regulated cellular pathways are supported and sustained by large amounts of ATP generated by the mitochondrial electron transport chain and oxidative phosphorylation, which is oxygen-dependent and is also the Achilles’ heel of the kidney, as the generation of reactive oxygen species (ROS) and oxidative stress is inevitable and may worsen under a variety of pathological conditions [4].

The kidney also undergoes an inevitable aging process. Indeed, it exhibits structural changes and functional decline even during a healthy aging process [5,6]. This is mainly manifested by a gradual decline of glomerular filtration rate (GFR), which has been estimated to be 0.7–0.9 mL/min/1.3 m^2^ on an annual basis [7]. While the mechanisms of this GFR decrease in healthy aging remain unclear, it has been associated with a progressive loss of nephrons starting from 30 years of age [7]. Clinically, this age-related decline in renal function is reflected by glomerulosclerosis, tubular atrophy, hyalinosis, loss of renal mass, arteriosclerosis, and interstitial fibrosis [5]. Age-related chronic inflammation may also be involved in this renal functional decline [6]. Collectively, all these slow pathological or physiological changes can eventually result in nephron hyperfiltration and hemodynamic stress, leading to increased sensitization of the kidney to a variety of insults such as sepsis, ischemia, drug toxicity, metal toxicity, and obesity, as well as diabetes [8,9,10,11,12]. Therefore, epidemiological studies have demonstrated that normal kidney aging can contribute to acute kidney injury (AKI) and chronic kidney disease (CKD) [10,13,14,15,16,17,18].

Given that kidney aging, like all aging affecting other organs in the body, is a complex and irreversible physiological process, there has been increasing interest in studying its mechanisms. In this respect, animal models of kidney aging have played an invaluable role in our understanding of how the kidney ages. Three animal models have been widely used for studying the aging kidneys, which involve natural aging rodent models [19,20], the senescence-accelerated mouse prone 8 (SAMP8) model [21], and D-galactose (D-gal)-induced aging in rodents. The natural aging model is time-consuming; the SAMP8 mouse model is a genetic model and is expensive and is limited to mice [5,22,23]. In contrast, the D-gal-induced aging model is economical, less time-consuming, and easy to establish [24,25]. In this review, we therefore focus on the D-gal model. This aging model has been widely used for the studies of the biological aging process and the evaluation of anti-aging strategies involving caloric restriction mimetics and the testing of numerous natural products and drugs [24,26,27,28,29].

## 2. D-Gal Catabolism and Common Mechanisms of D-Gal-Induced Aging

**Figure 1:**
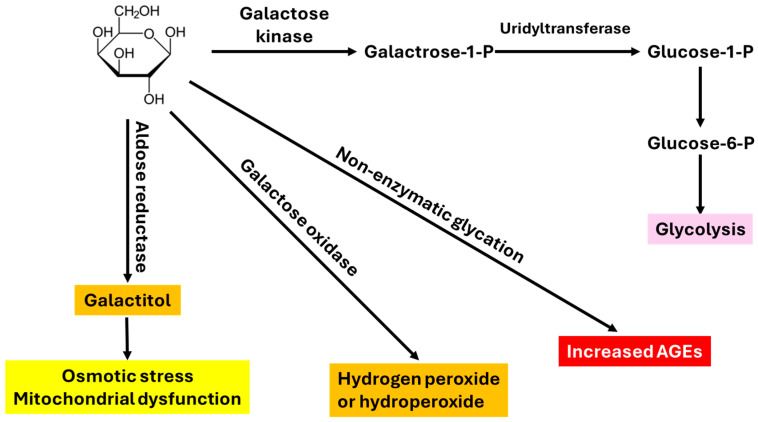
Catabolic pathways of D-galactose. Feeding into the glycolytic pathway is used to supply cellular energy by generating ATP. Metabolites such as galactitol, hydroperoxide, and advanced glycation end products (AGEs) resulting from the other three pathways all have detrimental effects on the kidney as well as other organs and tissues.

As opposed to natural aging, which is time-consuming, D-gal-induced aging is an accelerated process, which is convenient to perform within a short period of time [25]. Animals involved usually exhibit the least adverse effects and high survival rates [25]. D-gal is the third major monosaccharide of nutritional value, next to glucose and fructose [30]. When combined with glucose, it forms a disaccharide known as lactose, which exists in milk and dairy products. When compared with the structure of glucose, D-gal shows almost an identical structure except for a slightly different configuration at the C-4 position [30]. While being an energy supplier at normal concentrations in the body, high concentrations of D-gal are harmful to the body [31]. This is because at high levels, D-gal can form advanced end glycation products (AGEs) and can also be converted to hydroperoxide and aldose by galactose oxidase, leading to ROS generation [25,31]. Increased ROS production can cause oxidative stress, inflammation, fibrosis, mitochondrial abnormalities, and cell death [25,31]. It should be noted that a high level of galactose can also activate aldose reductase, leading to the conversion of galactose to galactitol, which can cause osmotic stress and mitochondrial dysfunction [32,33]. Deficiencies in galactokinase and uridyltransferase can also divert galactose conversion to galactitol [34]. Figure 1 outlines the D-gal catabolic pathways.

D-gal-accelerated aging in rodents was first discovered by Xu et al. in the 1980s [35]. Since then, D-gal has been extensively used for the study of aging and age-related degenerative disorders in a variety of animal organs [25]. It has also been used for aging studies in lower organisms such as Drosophila melanogaster [36]. Animals treated with D-gal show a shortened lifespan, a mitigated immune response, and declined cognition, with oxidative stress often being the common underlying mechanism of all the observed phenomena [24]. Furthermore, numerous aging markers have been found in D-gal-induced aging. These include increased AGEs and their corresponding receptors, telomere shortening, aldose reductase, sorbitol dehydrogenase, and senescence-associated β-galactosidase (SA-β-gal) [25,37,38]. β-amyloid, β-amyloid protein cleaving enzyme-1 and enhanced expression of senescence-linked genes have also been characteristics of D-gal-induced aging in a variety of animal models [25,39,40].

## 3. Kidney Aging Induced by D-Gal

D-gal treatment of animals capitulates many aspects of the aging process. These include increased oxidative damage to proteins, lipids, and DNA as reflected by increased protein carbonyl formation, lipid peroxidation products malondialdehyde, and DNA damage adducts 8-oxo-deoxy guanine (8-oxo-dG) [41,42,43,44]. Additionally, NADPH oxidases such as NADPH oxidase 4 (NOX4) [41], AGEs, and nitric oxide are all increased in conjunction with decreased antioxidant enzymes such as superoxide dismutase (SOD), catalase, glutathione peroxidase and glutathione reductase. Glutathione and total antioxidant capacities also decreased [45,46,47,48]. Moreover, kidney inflammation was enhanced following D-gal administration, which was reflected by up-regulation of signaling pathways, including NF-kB, iNOS, COX2, TNF-α, and IL-6 [48,49,50,51].

**Figure 2:**
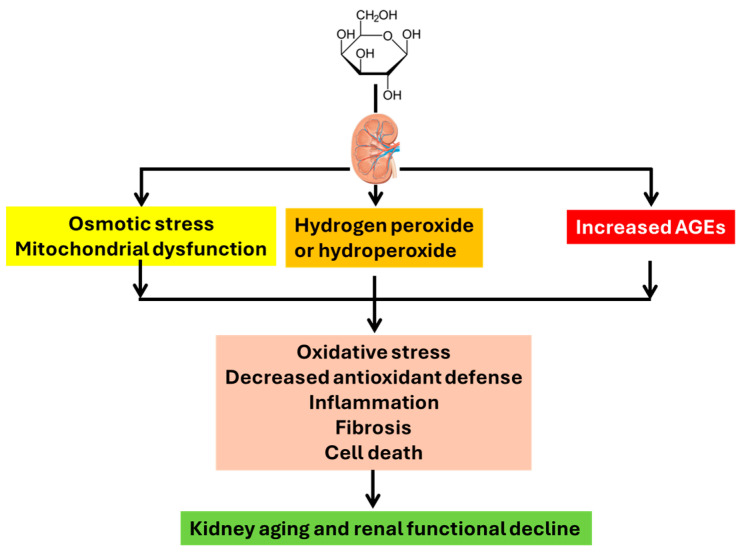
Common mechanisms by which D-gal induces kidney aging. These include oxidative stress, decreased antioxidant defense, inflammation, fibrosis, and cell death.

Decline in renal function after D-gal treatment was also obvious, as both blood urea nitrogen and creatinine increased while kidney index decreased [41]. Additionally, kidney injury markers such as cystatin C and uric acid contents also increased [41]. Structurally, histological analysis revealed lesions within the nephrons, including both glomerular damage and tubular damage. Glomerular structure analyzed by transmission electron microscopy indicated a thicker and uneven basement membrane induced by D-gal, demonstrating podocyte cell damage [47,51]. Figure 2 depicts the common mechanisms by which D-gal accelerates kidney aging. At this point, however, we would also like to briefly overview methods used to determine oxidative stress, inflammation, and fibrosis.

### 3.1. Analysis of Oxidative Stress

Determination of oxidative stress usually consists of three aspects: (1) measurement of ROS production, such as superoxide anion and H_2_O_2_; (2) measurement of oxidative damage to macromolecules such as proteins, DNA, and lipids; and (3) measurement of antioxidant capacities such as SOD, catalase, and glutathione.

#### 3.1.1. Measurement of ROS

ROS can be measured as the content of H_2_O_2_ [52] or by reduction in acetylated cytochrome c in an SOD-inhibitable manner [53]. ROS can also be measured by chemical probes such as 2,7-dichloroflurescin [54]. Additionally, total oxidant status can also be measured by a ferrous oxidation–xylenol orange assay [55,56,57].

#### 3.1.2. Measurement of Oxidative Damage to Macromolecules

This assessment includes protein oxidation, DNA oxidative damage, and lipid peroxidation. Protein oxidation can be measured as protein carbonyls derivatized by a probe called 2,4-dinitrophenylhydrazine (DNPH) [58]. DNP-derivatized proteins can be measured spectrophotometrically [59,60] or by Western blot assay using anti-DNP antibodies [61]. The advantage of the Western blot assay is that carbonylation of individual proteins in a given sample can be visualized. This contrasts with the spectrophotometric assay that measures total protein carbonyls in a given sample.

DNA oxidative damage can be measured by quantitation of 8-oxo-dG, a DNA adduct resulting from DNA oxidation [62]. 8-oxo-dG can be determined in many specimens, such as blood, urine, and tissue homogenates [63]. DNA fragmentation caused by oxidation can also be measured by a TUNEL assay, which is also used to measure the magnitude of apoptosis [64].

Lipid peroxidation can be measured by quantitation of either malondialdehyde (MDA) or 4-hydroxynonenal (HNE) [60,65]. The latter can also be analyzed as a protein conjugate by Western blot assay using anti-HNE antibodies [65].

#### 3.1.3. Measurement of Antioxidant Defense System

Cellular antioxidant capacities can be measured by a variety of parameters. These include activities of antioxidant enzymes such as superoxide dismutase, catalase, glutathione peroxidase, peroxiredoxins and thioredoxins [66,67,68]. Glutathione, the major cellular antioxidant [69,70], is often measured in conjunction with the determination of antioxidant enzymes. Additionally, total antioxidant capacity can also be quantified to reflect overall cellular antioxidant status [71,72,73].

#### 3.1.4. Measurement of Inflammation and Fibrosis

Determination of inflammation can be conducted by measuring pro-inflammatory cytokines, such as IL-6 and IL-1β [74,75]. The level of TNF-α and activation of the NF-kB signaling pathway can also be measured to indicate the magnitude of inflammation [75,76,77]. For analysis of tissue fibrosis, accumulation of collagen and extracellular matrix proteins can often be measured to indicate the magnitude of fibrosis [78,79,80].

In addition to the common mechanisms, such as oxidative stress, inflammation, and fibrosis, discussed above, certain specific mechanisms have also been found to contribute to D-gal-induced kidney aging. For example, ferroptosis, iron-mediated cell death, has been reported to be linked to D-gal-induced kidney aging via promotion of tubular senescence [81]. Table 1 summarizes some uncommon but outstanding mechanisms by which D-gal accelerates kidney aging.

### 3.2. General Experimental Approaches of D-Gal-Induced Aging in Rodents

**Figure 3:**
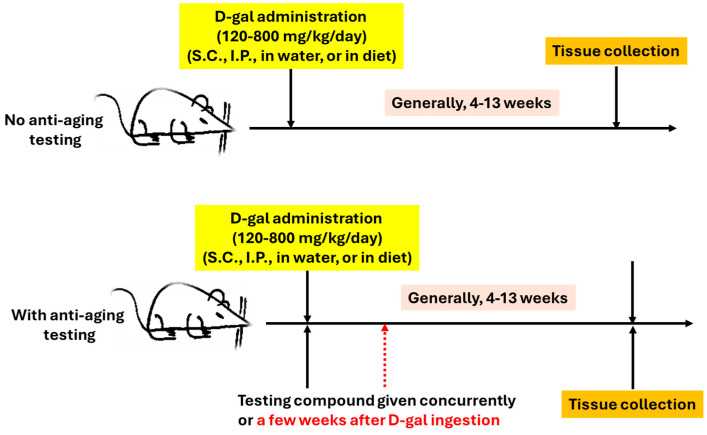
General protocols used for D-gal-induced overall aging or kidney aging, as well as the testing of anti-aging treatments.

In the literature, experimental protocols reported, among others, kidney aging studies for D-gal-accelerated aging in mice or rats vary widely in terms of dosage, route of administration, and duration of induction. Different laboratories usually have different protocols. The dosage of D-gal can range from 100 mg/kg/day to 1000 mg/kg/day. However, most investigators use a dosage between 120 mg/kg/day and 800 mg//kg/day (see Table 1 and Table 2). With respect to D-gal administration, the most used route of ingestion is via subcutaneous injection. Intraperitoneal injections have also been conducted by some investigators. Moreover, D-gal in drinking water ranging from 5% to 40% (*w*/*v*) [87,88] and in a solid diet ranging from 30 to 50% (*w*/*w*) [89,90] have also been used. For the duration of D-gal induction, 4 weeks to 13 weeks have been reported. When anti-aging compounds were tested, a compound could be given concurrently or a few weeks after D-gal ingestion was started. Nonetheless, for a given individual laboratory, the protocol usually remained the same so that results from study to study could be compared. Figure 3 shows the general protocols.

## 4. D-Gal-Induced Aging Model as a Platform for Evaluating Anti-Kidney Aging Strategies

Similar to any other animal model, D-gal-induced aging in rodents has also been widely used for testing strategies designed for anti-kidney aging purposes. Among these strategies, many are natural products such as polyphenols, plant extracts, chemicals, and pharmacological drugs. Table 2 lists various agents that have been evaluated. D-gal dosage, route of administration, and duration of the experiments are also given. Numerous agents show common anti-kidney aging mechanisms, including counteracting oxidative stress, mitigating inflammation, and attenuating renal fibrosis, as well as increasing autophagy or mitophagy [82,91,92].

**Table 2:** Potential effects of various agents, such as plant natural products, pharmacological drugs, and chemicals, on kidney aging induced by D-galactose.

Chemical/Compound/ Drug/Approach/Species	D-Gal-mg/kg/day Duration/Route	Mechanism of Action	Reference
Exercise/Rat	100, I.P., 9 weeks	Antioxidative stress	[93]
Que Zui tea/Mouse	300, S.C., 10 weeks	Antioxidative stress	[94]
Vitamin E/selenium/carrot anthocyanins	400, I.P., 6 weeks	Antioxidative stress	[50]
Silk sericin/Mouse	250, I.P., 60 days	Antioxidative stress	[95]
Gemfibrozil/Mouse	150, I.P., 6 weeks	Antioxidative stress	[96]
Calcium dobesilate/Mouse	500, P.O., 6 weeks	Antioxidative stress	[97]
Sulforaphene/Mouse	300–800, S.C. varying	Antioxidative stress	[98]
Quercetin/Rat	120, S.C., 6 weeks	Antioxidative stress	[99]
Korean red ginseng/Rat	100, I.P. 8 weeks	Antioxidative stress	[8]
Ginsenoside Rg1/Mouse	120, S.C., 6 weeks	Antioxidative stress	[48]
20(R)-ginsenoside Rg3/Mouse	800, S.C., 8 weeks	Antioxidative stress	[100]
Rosa roxburghii tratt glycosides, quercetin/Mouse	100, S.C, 6 weeks	Antioxidative stress	[101]
Fucoidan oligosaccharide/Rat	150, S.C., 8 weeks	Antioxidative stress	[92]
Arctium lappa L. polysaccharides/Mouse	150, I.P., 8 weeks	Antioxidative stress	[102]
Antarctic ice microalgae polysaccharides/Mouse	120, I.P., 6 weeks	Antioxidative stress	[103]
Chitosan oligosaccharide/Mouse	250, S.C., 8 weeks	Antioxidative stress	[104]
Small-leaved Kuding tea polyphenols/Mouse	120, I.P., 6 weeks	Antioxidative stress	[105]
*Apocynum venetum* polyphenols/Mouse	120, I.P., 6 weeks	Antioxidative stress	[106]
Radix isatidis protein/Mouse	800, S.C., 7 weeks	Antioxidative stress	[107]
Collagen polypeptide	300, S.C., 8 weeks	Antioxidative stress	[108]
Centella asiatica/Rat	60, I.P., 70 days	Antioxidative stress	[109]
Dendrobium nobile alcohol extract/Mouse	125, S.C., 8 weeks	Antioxidative stress	[110]
Ethyl acetate extraction from *Idesia polycarpa*	100, S.C., 6 weeks	Antioxidative stress	[111]
Dendrobium officinale extract/Mouse	120, S.C., 8 weeks	Antioxidative stress	[112]
Angelica sinensis extract	200, S.C., 8 weeks	Antioxidative stress	[49]
Kaempferia parviflora extract	50, I.P., 60 days	Antioxidative stress	[113]
Alpinate oxyphyllae fructus/Rat	150, N/A, 6 weeks	Antioxidative stress/anti-inflammation	[114]
Chlorogenic acid/Mouse	100, S.C., 8 weeks	Antioxidative stress/anti-inflammation	[47]
Urolithin A/Mouse	150, S.C., 8 weeks	Antioxidative stress/anti-inflammation	[115]
Ginsenoside Rg1/Mouse	120, I.P., 7 weeks	Antioxidative stress/anti-inflammation	[116]
Artemisia annua L extract	100, S.C., 6 weeks	Enhancing antioxidant defense	[117]
Selenoarginine/Mouse	150, S.C., 6 weeks	Enhancing antioxidant defense	[118]
Lactobacillus brevis/Mouse	300, S.C., 5 weeks	Enhancing antioxidant defense	[119]
Rhein lysinate/Mouse	100, S.C., 8 weeks	Enhancing antioxidant defense	[120]
Moringa Oleifera see aqueous extract/Rat	30% in water, 4 weeks	Enhancing antioxidant defense	[88]
Lithium chloride/Rat	300, I.P., 6 weeks	Enhancing antioxidant defense	[121]
Box A of HMGB1/Rat	150, S.C., 8 weeks	Inhibiting DNA damage	[122]
Troxerutin/Mouse	500, S.C., 8 weeks	Inhibiting DNA damage	[41]
Klotho/Mouse	500, S.C., 6 weeks	Inhibiting DNA methyltransferase	[123]
L-theanine/Rat	200, S.C., 8 weeks	Decreasing AGEs accumulation	[37]
Vitamin B12/Rat	300, I.P., 120 days	Decreasing AGEs accumulation	[38]
Resveratrol/Mouse	1000, S.C.,8 weeks	Decreasing AGEs accumulation	[124]
Exogenous hydrogen sulfide/Mouse	150, S.C., 10 weeks	Suppressing mitochondrial dysfunction	[91]
β-catenin inhibitors, KYA1797K/Mouse	150, S.C., 6 weeks	Suppressing mitochondrial dysfunction	[125]
Resveratrol/Mouse	1 mg/kg, I.P., 4 weeks	Increasing klotho expression	[126]
Ganoderma lucidum/Mouse	600, S.C., 8 weeks	Increasing klotho expression	[127]
Moderate beer consumption/Mouse	25, S.C., 8 weeks	Modulating gut microbiota	[128]
Polygonatum sibiricum polysaccharides/Mouse	150, I.P., 8 weeks	Modulating gut microbiota	[129]
Astaxanthin/exercise/Rat	100, I.P., 6 weeks	Nrf2 activation	[130]
Alginate oligosaccharide/Mouse	200, S.C., 8 weeks	Nrf2/NQO1/HO-1 activation	[43]
Black rice anthocyanins	500, S.C., 13 weeks	Nrf2/NF-kB signaling	[131]
Tropisetron/Mouse	200, S.C., 8 weeks	Sirt1 up-regulation	[42]
Piperlongumine 1–3/Mouse	500, I.P. 10 weeks	Sirt1 up-regulation	[132]
Mild moxibustion/Rat	300, I.P., 4 weeks	Sirt1/p53 signaling	[133]
Zuoguiyin (traditional Chinese medicine)/Rat	125, S.C., 8 weeks	Sirt1/PPARγ signaling	[134]
Maltol/Mouse	800, I.P., 7 weeks	p53/p21/p16, PI3K/Akt pathways	[135]
Adropin/vitamin D/Rat	120, I.P., 8 weeks	MAPK/HIFα/VEGF/eNOS	[136]
Serum-free, adipose conditioned medium/Mouse	100, S.C., 8 weeks	Suppressing IL-6/STAT3	[137]
Exercise/Rat	150, S.C., 8 weeks	Suppressing SGLT2 expression	[138]
*Vitex agnus-castus* extracts	500, S.C., 45 days	Suppressing apoptosis	[139]
Methyltransferase-like protein 3/Mouse	500, S.C., 8 weeks	Promoting miR-181a maturation	[140]
Lycopene/Mouse	150, I.P., 8 weeks	Improving insulin signaling	[141]
Daytime-restricted feeding/Mouse	100, I.P., 16 weeks	Decreasing renal damage	[142]

## 5. Miscellaneous Applications of the D-Gal Aging Model

### 5.1. Synergistic Detrimental Effects with Risk Factors

D-gal-induced acceleration of aging can be accentuated by other disease-causing risk factors, such as high-fat-diet-induced obesity and arsenite toxicity. For example, Park et al. [8] reported that when an aging rat was fed with a high-fat diet, both oxidative stress and non-enzymatic protein glycation were further increased when compared with those of only D-gal induction. Moreover, DNA damage, nephron apoptosis, and extracellular high mobility group box 1 were also additionally elevated, demonstrating aggravation of D-gal-induced kidney aging by a high-fat diet. Similarly, Akbari et al. [143] reported that when a D-gal-induced aging rat was treated with sodium arsenite, oxidative damage, down-regulation of antioxidant defense, and cell death were all accentuated by arsenite on top of D-gal-induced aging. It should be noted that this study was focused on the testis instead of the kidney. Nonetheless, it is conceivable that the kidney would exhibit a similar observation to that of the testis. Taken together, these studies demonstrate that D-gal-induced aging could be worsened by other disease-causing risk factors.

### 5.2. D-Gal-Induced Aging as a Platform to Test the Anti-Aging Effects of Caloric Restriction Mimetics

Caloric restriction (CR) is a proven approach that can delay aging and the age-related onset of diseases [144]. The underlying mechanisms of CR have been thought to involve modulation of mTOR and AMPK signaling pathways, oxidative stress, inflammation, mitochondrial abnormalities, and the NAD^+^-dependent signaling pathway [145,146,147,148,149,150]. Given that CR has an incompliance issue in humans, numerous researchers have focused on CR mimetics, in hopes that such mimetics can produce the same beneficial effects as CR. While no studies have been conducted exclusively on the kidney in terms of CR mimetics and D-gal-induced kidney aging, many studies have been performed on overall aging or on other organs using the D-gal aging platform. For example, chrysin, a glycolytic pathway inhibitor, has been shown to mimic CR in a rat model of D-gal aging [151]. It was found that chrysin inhibited all the D-gal-induced aging biomarkers, such as protein oxidation, lipid peroxidation, and protein glycation. Moreover, chrysin also significantly up-regulated the antioxidant defense system and down-regulated the inflammatory response. Similarly, chitosan, a polysaccharide, has also been shown to exhibit CR effects in D-gal-induced rat aging [28]. Table 3 lists CR mimetics that have been tested on the D-gal aging platform.

### 5.3. Potential Difference Between D-Gal Oral Intake vs. D-Gal Injection

Martinovic et al. [159] recently reported a potential difference between chronic oral intake of D-gal and I.P. or S.C. D-gal injections. The authors used male Wistar rats and addressed a systematic oxidative stress issue upon chronic oral intake of D-gal that was administered at 200 mg/kg and 500 mg/kg, respectively. The effects of oral intake were also compared with those of natural aging in rats. It was found that oxidative stress was elevated in the heart, liver, and kidney, and this elevation was similar to that observed in 30-month-old rats. In contrast to pronounced oxidative stress, only minor histopathological lesions were detected with nearly normal organ function. Taken together, the authors suggest that chronic oral intake of D-gal only imposes milder effects on organs and may induce only certain features of natural aging. Therefore, to mimic a significant aging process, D-gal administration via S.C. or I.P. injections should be performed.

### 5.4. Effects of Ketone Bodies and Ketogenic Diet

Ketone bodies can provide an alternative source of energy to the brain and other peripheral organs under prolonged fasting or starvation conditions [160,161]. They refer to three metabolic products, namely β-hydroxybutyrate, acetoacetate, and acetone [4]. Acetone is usually exhaled from the lungs and thus cannot be used as an energy source [3]. Ketone bodies are produced in the liver due to low carbohydrate intake in the body, which is usually triggered by a high glucagon-to-insulin ratio [4]. Under this condition, fatty acids are overburned, leading to excessive acetyl-CoA production that overwhelms the TCA cycle. Surplus acetyl-CoA is thus diverted for ketone body production [4]. Endogenous ketone bodies, particularly β-hydroxybutyrate, can also be highly enriched by ingestion of a ketogenic diet or just β-hydroxybutyrate and its derivatives [162,163]. Ketone bodies have been shown to have renoprotective properties under a variety of renal pathological conditions, including AKI, CKD, and DKD [164,165,166,167]. However, the potential effects of ketone bodies on D-gal-induced kidney aging are yet to be comprehensively evaluated. Nonetheless, given that ketone bodies can protect the kidneys against numerous insults, it is conceivable that ketone bodies may show anti-aging effects in the D-gal-induced kidney aging model.

### 5.5. Identification of Common Targets for Both Kidney Aging and CKD

Kidney aging increases the occurrence of CKD [6,7,18]. Therefore, the two processes may have common targets that can be explored for slowing down both kidney aging and the development of age-related CKD, such as DKD [168]. Guo et al. recently reported such a potential target [11]. The authors found that AMP-activated protein kinase-related kinase-1 (NUAK1) was up-regulated in both D-gal-induced kidney aging and HFD/streptozotocin-induced DKD. Down-regulation of NUAK1 via gene expression manipulation ameliorated nephron senescence and DKD, and the underlying mechanism by which NUAK1 contributed to kidney aging and DKD involved the ROS/p53 axis. The authors also discovered that asiatic acid [169,170], a traditional herbal element, could physically bind to NUAK1 and inhibit its activities, thereby achieving a therapeutic purpose. This study also provides a novel strategy that can be adapted to further identification of more common targets that may be used to combat both kidney aging and CKD, including DKD.

## 6. Conclusions and Future Perspectives

The natural aging process in rodents can be accelerated by D-gal administration. Here in this review article, we discussed this animal model of aging with a focus on kidney aging. Common mechanisms of D-gal-induced kidney aging include oxidative stress, inflammation, fibrosis, and cell death. In many studies, down-regulation of the antioxidant defense system has also been demonstrated. Collectively, these pathological mechanisms can cause functional decline in the kidney, which can also be augmented by other disease-causing risk factors such as obesity and environmental metal toxicity. This animal model of kidney aging has also been widely used as a platform to test the anti-aging properties of numerous natural products, chemicals, and drugs. It has also been used to evaluate the anti-aging properties of many caloric restriction mimetics. It should be noted that different researchers often differ on D-gal dosages, route of administration, and duration of aging induction. This may create issues when attempting to compare results from different laboratories. Nonetheless, this model will continue to lend its convenience and usefulness to the study of kidney aging mechanisms and to the evaluation of potential anti-kidney aging strategies.

## Figures and Tables

**Table 1:** Uncommon and specific mechanisms involved in D-gal-induced kidney aging.

Animal Models	D-Gal Dosage (mg/kg/day)/ Route/Duration	Specific Mechanisms	Reference
Mouse	300, S.C., 8 weeks	Ferroptosis	[81]
Mouse	500, S.C.,12 weeks	Caveolin-1 up-regulation	[82]
Mouse	500, S.C., 8 weeks	M1 macrophage polarization	[83]
Mouse	150, I.P., 8 weeks	BRD4 up-regulation	[84]
Mouse	150, S.C., 8 weeks	Enhanced TRPC3 transcription	[85]
Mouse	150, S.C., 6 weeks	Cannabinoid receptor 2 up-regulation	[86]

**Table 3:** Caloric restriction mimetics have been tested using the D-gal aging induction animal model.

CR Mimetics	Reference
Chrysin	[151]
Fisetin	[27]
Spermidine	[152]
Curcumin	[153]
Chitosan	[28]
Glucosamine	[154]
2-Deoxy-d-glucose	[155]
Metformin	[156]
Goat milk	[157]
Caloric restriction per se	[158]
Daytime-restricted feeding	[142]

Note: these studies were not specifically on the kidneys.

## Data Availability

No new data were created or analyzed in this study.
